# Can we improve therapeutic compliance in cancer patients? Report of a cross-sectional case series study in Morocco

**DOI:** 10.1097/MS9.0000000000001869

**Published:** 2024-03-21

**Authors:** Mokhtari Khadija, Hajji Bekkay, El oualy Hanane, Madani Hamid

**Affiliations:** a Laboratory of Energy, Embedded System and Information Processing, National School of Applied Sciences, Mohammed First University, Oujda, Morocco; b Laboratory of Medical Oncology, Faculty of Medicine and Pharmacy, Mohammed First University, Oujda, Morocco

**Keywords:** cancer, compliance, oncology, patient, therapeutic

## Abstract

Therapeutic compliance (TC) refers to the patient's compliance with the prescriptions and recommendations of a doctor. Patients with cancer often exhibit unsatisfactory TC. The objective of our study was to assess TC levels in cancer patients and identify predictors of poor compliance. The authors conducted a cross-sectional study in March 2023 at the oncology centre, where the vast majority of medical activity is performed in the day hospital. TC was measured using a questionnaire or survey. Various parameters were analyzed to identify predictive factors of poor therapeutic compliance. The authors’ study included 175 cancer patients with a mean age of 55 years. The study revealed that 85% exhibited good compliance (GC) as indicated by the CI [8.500 ± 0.075], signifying patients who consistently adhered to their medication schedule. Conversely, 15% demonstrated poor compliance (PC), as indicated by the confidence interval [0.825 ± 0.750]. Compliance status was assessed by considering patients who adhered to the prescribed medication timing as good compliance (GC) and those who did not adhere as poor compliance (PC). The analysis of our study results indicated that poor therapeutic compliance was associated with low socio-economic and educational levels. Therefore, it is important to utilize all available resources to improve therapeutic compliance. The majority of factors contributing to poor compliance can be mitigated through effective coordination between the patient and their support network.

## Introduction

Highlights

- This study underscores the significance of coordination between patients and their families to enhance therapeutic compliance, emphasizing a critical aspect both in practical and social terms.
- A comparison of the results in our study with those documented in the literature reveals.
- The potential use of innovative technological tools, such as electronic pill dispensers, to tackle compliance issues is explored. This highlights an ongoing search for inventive solutions.
- The study recognizes the influence of socio-economic and cultural factors on therapeutic compliance, emphasizing a holistic approach that considers various influences on patient behaviour.

Similar to many developing countries, the International Agency for Research on Cancer (IARC) reports a cancer mortality rate of 86.9 per 100000 in Morocco^1^. This poses a significant public health challenge, impacting not only the quality of life for patients but also leading to economic consequences due to inadequate disease control. The gravity of the situation underscores the importance of addressing factors influencing therapeutic compliance. Therapeutic compliance, defined as the alignment between a patient’s behaviour and their doctor’s recommendations, is a crucial parameter in the therapeutic management of cancer patients^2^. Poor compliance is a pervasive issue in most chronic diseases, contributing to increased morbidity, mortality, and healthcare expenditures with broad epidemiological and economic implications^3^.

Despite the high prevalence of cancer in Morocco, data on therapeutic compliance, which could substantiate the impact on morbidity and mortality, is notably scarce. It is vital to recognize that therapeutic compliance, the counterpart to therapeutic compliance, is an established concept in internal medicine^4^. However, in oncology, it remains a relatively novel concept, closely tied to the rising use of new oral formulation treatments. Adhering meticulously to a doctor’s prescriptions and adhering to hygiene-dietary rules are integral to achieving optimal therapeutic benefits. This study aims to assess the level of therapeutic observance and identify potential predictive factors associated with poor therapeutic observance in cancer patients under the care of the Regional Oncology Center. By delving into these factors, we hope to contribute valuable insights for improving the overall management of cancer patients in the region.

## Methods

### Study design

This research employs a descriptive, analytical, and cross-sectional study design conducted among cancer patients receiving care at the Regional Oncology Center, Morocco. This case series has been reported in accordance with the PROCESS guidelines^5^.

### Settings and timeframes

The data collection phase encompassed various departments, including the day hospital, radiotherapy, surgery department, palliative care, and the Oncology Pharmacy at the Regional Oncology Center. Throughout the study, obtaining patient consent was a mandatory step, and the research protocol ensured that participants were not exposed to any additional risks.

### Study population

All participants in our study were diagnosed with cancer and underwent treatment involving at least one orally administered drug. The survey was conducted over 60 days, commencing on 1 March 2023, and concluding on 1 May 2023.

The determination of the sample size was achieved through the utilization of a single population proportion formula. Considering a proportion of 15%, a CI of 95%, and a margin of error of 5%, the calculated sample size amounted to 175 participants. Consequently, the study aimed to include a minimum of 175 patients to ensure statistical robustness and reliability.

### Data collection

Patients meeting the eligibility criteria were enroled during their medication appointments. Information was collected through a questionnaire based on the oral responses of the patients, which was divided into three parts: the first included the identification of the patient (age, sex, city, socio-economic level, level of learning, social regime), the second part consisted of questions relating to the doctor-patient, and the third part consisted of questions relating to the evaluation of therapeutic observance (Do patients commit to taking their medication on time? And do they use memory aids to remember when to take their medication?). The questionnaire comprised a total of 26 items, consisting of eight closed questions, eleven multiple-choice questions, and the remaining questions requiring short answers.

At the end of the inclusion period, the data was entered into Google Forms, and the calculations were made online using the Check Market web application.

### Description of the variables studied

Patients admitted to the oncology centre all have pathological evidence or imaging results suggestive of progressive cancer. Socio-demographic characteristics, age, sex, level of education, and professional situation, as well as treatment characteristics, disease duration, associated diseases, and prescribed medications, were obtained from patient reports.

### Evaluation of therapeutic compliance

Oral therapies in oncology play a crucial role in the management of cancerous pathology^6^. Evaluating therapeutic compliance can be accomplished through various methods, ranging from simple to more sophisticated approaches. Some of these means include:

- Simple, non-inquisitive questioning gives the patient confidence so that he can make an objective assessment of the follow-up of his treatment.
- Control of the renewal of prescriptions (possibly with the assistance of the pharmacist).
- Visualization of home pharmacy cabinets.

In clinical trials, the two most widely used methods are counting the remaining tablets (“pill-count”) and, more recently, the use of electronic pill dispensers, which record and date each bottle opening^7^.

In our study, we opted to judge the level of therapeutic compliance in patients with simple questioning.

## Results

### Participants

During this study, data were collected from 175 patients undergoing treatment at the oncology centre. We acknowledge the limited sample size in our article, emphasizing its potential impact on result generalization and the need for further research with larger cohorts. The patients had a mean age of 55 years, with a median age of 57 years. Notably, 56.57% of the participants were aged 41 years or older. The study population exhibited a clear female predominance (78%) (confidence interval: [7.724; 7.875]). These demographic details offer insights into the age distribution and gender composition of the patient cohort in the study.

### Outcomes and follow-up

There was no deviation from the initial management plans. The majority of our patients had a low level of education: 69.71% were illiterate, 26.28% had a primary education level, 2.85% had a medium or secondary education level, and 1.14% had a university level. The socio-economic level of the majority of our patients was quite low (98.28%). Most of the patients had an AMO TADAMONE 98.85% social diet (Table 1).

**Table 1:** Socio-demographic characteristics of 175 cancer patients.

Characteristic	Effective (*n*)	(%)
Sex		
Female	138	78.85
Male	37	21.14
Age group (years)		
Under 40	12	6.8571
Between 41 and 61	99	56.5714
Between 61 and 81	59	33.714
Greater than 81	5	2.8571
Educational level		
Illiterate	122	69.71
Primary	46	26.28
Medium, secondary	5	2.85
University	2	1.14
Socio-economic level		
Low	172	98.28
Medium	3	1.71
High	0	0
Type of social scheme		
AMO TADAMONE	173	98.8%
CNOPS	2	1.14
CNSS	0	0

CNOPS: National Fund of Social Security organizations,

CNSS: The National Social Security Fund.

AMO TADAMONE: Compulsory Health Insurance is a plan that guarantees a basket of care identical to that of employees.

The majority of patients had a single drug in their oral treatment, and 33.14% had a single treatment. 10.28% of cases had diabetes, and 5.4% had hypertension (Table 2).

**Table 2:** Disease and treatment-related characteristics of 175 patients.

	Effective (*n*)	(%)
Oral treatment		
A single drug	151	86.28
Two or more drugs	24	13.71
Other therapies (non-oral)		
One treatment	58	33.14
Two or more treatments	117	66.85
Associated diseases		
Hypertension	9	5.14
Diabetes	18	10.28
Other	5	2.85

The majority of patients had mobile phones (81.71%), 51.42% had smartphones, and 46.28% had an internet connection (Table 3).

**Table 3:** Characteristics related to the means available (telephone, connection, etc.) in 175 patients.

	Effective (*n*)	(%)
People who have a mobile phone	143	81.71
people who have a Smartphone	90	51.42
People who have internet connection	81	46.28
People who have internet connection every day	61	34.85

**Figure 1:**
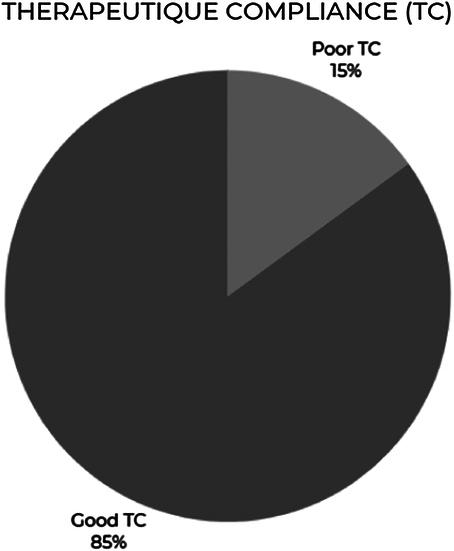
The rate of therapeutic compliance.

The therapeutic compliance rate revealed 85% good compliance and 15% poor compliance (Fig. 1).

Poor therapeutic compliance (PTC) is directly proportional to the level of education (Illiterate 14.85%). Also, the PTC is related to the socio-economic level (low 20.57%). The possession of technological means (mobile phone 42.28%, Smartphone 42.85%, internet connection 34.85%) strongly contributes to good therapeutic observance (Table 4).

**Table 4:** Analysis of therapeutic compliance rate.

Setting	Good therapeutic compliance, *n* (%)	Poor therapeutic compliance, *n* (%)
Educational level (Illiterate)	96 (54.85)	26 (14.85)
Socio-economic level (low)	136 (77.71)	36 (20.57)
Mobile phone	74 (42.28)	69 (39.42)
Smartphone	75 (42.85)	15 (8.57)
Internet connection	61 (34.85)	20 (11.42)

### Adherence and compliance

The patient respected the appointment to take the medications; this was confirmed directly with the patient and the manager of the oncology pharmacy.

## Discussion

Non-compliance with treatment is a widespread issue, even among cancer patients, posing a significant barrier to achieving clinical and therapeutic objectives in both emerging and developed countries. The primary goal of any prescribed medical therapy is to achieve desired outcomes in patients, which is integral to effective disease management. However, poor therapeutic compliance jeopardizes the potential for these outcomes, posing high risks for life-threatening consequences^4^.

Our study reveals that non-adherence to treatment in cancer patients is a frequent and multifactorial phenomenon influenced by various factors, including education level, socio-economic status, and the absence of technological means (mobile phones, smartphones, internet connection, etc.). Discrepancies between our results and other studies can be attributed to differences in socio-economic and cultural levels among the study samples.

In Morocco, a survey on therapeutic observance in colorectal cancer patients identified socio-economic factors and education level as contributors to poor observance^8^. Similarly, our study underscores the significant impact of low socio-economic levels and educational backgrounds on poor therapeutic compliance.

Numerous studies, including ours, have established a direct correlation between low education levels and poor therapeutic compliance. Patients with limited cultural understanding often struggle to comprehend the importance of prescribed treatments^9^. Moreover, good therapeutic compliance is closely tied to technological means, where patients equipped with such technology can more effectively manage medication schedules, leveraging features like alarms on their mobile phones to prompt timely medication intake.

Challenges associated with adhering to medical prescriptions were highlighted in a 1994 survey by the French Committee for Health Education, revealing not only the prevalence of self-medication (nearly 60% of cases) but also frequent modifications to medical prescriptions, including adjustments to therapeutic doses and treatment durations^10^.

In oncology, non-optimal compliance is associated with high recurrence rates and increased mortality. These outcomes are scrutinized in ambulatory care, allowing healthcare professionals to assess the impact of their follow-up on patient compliance^11^. A 2003 WHO report identified poor compliance with long-term treatment for chronic diseases as a leading cause of the diminished effectiveness of care worldwide.

In France, a 2012 study by Fabien Despas and Henri Roche underscored the oversight of compliance with prescribed therapies, finding particularly poor compliance, especially in cases where drugs were administered orally over an extended period^12^. A 2016 study by the ARC (Foundation for Cancer Research) revealed that compliance with hormone therapy treatment for women affected by breast cancer was estimated to be only 50–65% after 5 years, highlighting ongoing challenges in this aspect of cancer care.^13^

A study carried out by Pierre Nizet in 2021 emphasized the need to support patients in following up on their treatment so that they understand it, take ownership of it, and adhere to it through an exchange of views and an active listening posture in order to provide them with information tailored to their needs, taking into account their stage of acceptance of the disease and their level of literacy. This emphasizes the important role of patients’ literacy level for successful therapeutic compliance.^14^

Acknowledging the inherent limitations from our relatively small sample size of 175 patients, our findings provide valuable insights into factors influencing therapeutic compliance among the specific group studied at the oncology centre. However, caution should be exercised when extending these conclusions to the entire population of cancer patients in the country.

The demographic characteristics of our study population, including age distribution and sex composition, may not fully represent the diversity in the broader cancer patient population. The study primarily focused on patients undergoing treatment at the oncology centre, and therefore, the results may not capture variations in therapeutic compliance observed in different cancer types, stages, or treatment settings.

Furthermore, the clear female predominance (78%) in our study raises questions about the generalizability of our findings to male cancer patients. This sex imbalance within our sample suggests a potential limitation in extrapolating our results to the entire population without considering gender-specific factors influencing therapeutic compliance.

To enhance the external validity of our findings, future research should aim for larger and more diverse samples encompassing various cancer types, stages, and treatment contexts. Such efforts will contribute to a more comprehensive understanding of the factors affecting therapeutic compliance across the entire spectrum of cancer patients in the country. While our study provides valuable insights within the confines of our sample, cautious interpretation is warranted when applying these findings to the broader population of cancer patients.

### Limits

Our study has several limitations. Being designed as an exploratory study, it was conducted at a single hospital with a relatively small sample size of 175 patients. Additionally, the respondents’ profile is highly diverse, encompassing variations in age, types of cancer, socio-economic characteristics, and more. This diversity may limit the generalizability of the results to a broader population of cancer patients in the country. It’s essential to recognize that the patient perceptions presented in this study cannot fully encapsulate the wide range of opinions among cancer patients.

Certain elements, such as the limited sample size and specific characteristics of respondents, may impact the applicability of the results. Therefore, the results presented here are considered hypothetical and should serve as a basis for designing actions to improve patient support.

Moreover, it’s worth noting that the majority of participants had previously undergone intravenous chemotherapy. This previous exposure may have influenced the limited number of individuals visiting the oncology pharmacy for medication, and, as a result, we did not have the opportunity to conduct the survey with them. Further research is needed to explore the nuances of this influence and its implications for medication adherence in cancer patients.

## Conclusion

In conclusion, our study underscores the critical role of therapeutic education and psychosocial support in empowering patients to confront cancerous diseases. The challenge of treatment compliance among individuals with cancer emphasizes the urgency for proactive measures. Addressing the factors contributing to poor compliance necessitates effective coordination between the patient and their support network. Furthermore, the integration of innovative technological tools, such as electronic pillboxes, holds promise in mitigating compliance issues and enhancing patient adherence to treatment plans. Through collaborative efforts, educational initiatives, the adoption of technological advancements, and fostering of research partnerships between healthcare institutions, research organizations, and technology companies, substantial progress can be made in overcoming the obstacles associated with therapeutic compliance in the context of cancer care.

## Ethical approval

Formal approval from the ethics committee was deemed unnecessary for this case series. Instead, each participant offered their verbal informed consent following a thorough explanation of the study’s nature, and prior to any involvement. Participation in the study was entirely voluntary, and individuals had the freedom to decline if they chose not to take part.

## Consent

Oral informed consent was obtained from the patient for publication of this case series.

## Patient perspective

After explaining to the patient about the entire project and its importance in the lives of patients he extremely agrees to participate.

## Source of funding

None declared.

## Author contribution

All authors were involved in the writing of this article. The case is not presented at a conference or regional meeting.

## Conflicts of interest disclosure

The authors declare that they have no conflicts of interest.

## Research registration unique identifying number (UIN)

Our paper is a case series; no registration was done for it.

## Guarantor

Khadija Mokhtari.

## Data availability statement

Data sharing is not applicable to this article.

## Provenance and peer review

Not commissioned, externally peer-reviewed.
